# CNN-LSTM-Based Nonlinear Model Predictive Controller for Temperature Trajectory Tracking in a Batch Reactor

**DOI:** 10.1021/acsomega.4c07893

**Published:** 2024-11-12

**Authors:** Aishwarya Selvamurugan, Parthiban Kunnathur Ganesan, Shashank S Nayak, Arockiaraj Simiyon, Thirunavukkarasu Indiran

**Affiliations:** † Computer Science Engineering, Sri Eshwar College of Engineering, Coimbatore 641202, Tamil Nadu, India; ‡ Department of Biomedical Engineering, Dhaanish Ahmed Institute of Technology, Coimbatore 641105, Tamil Nadu, India; § Manipal School of Information Science, Manipal Academy of Higher Education, Manipal 576 104, Karnataka, India; ∥ Department of Instrumentation and Control Engineering, Manipal Institute of Technology, Manipal Academy of Higher Education, Manipal 576 104, Karnataka,India

## Abstract

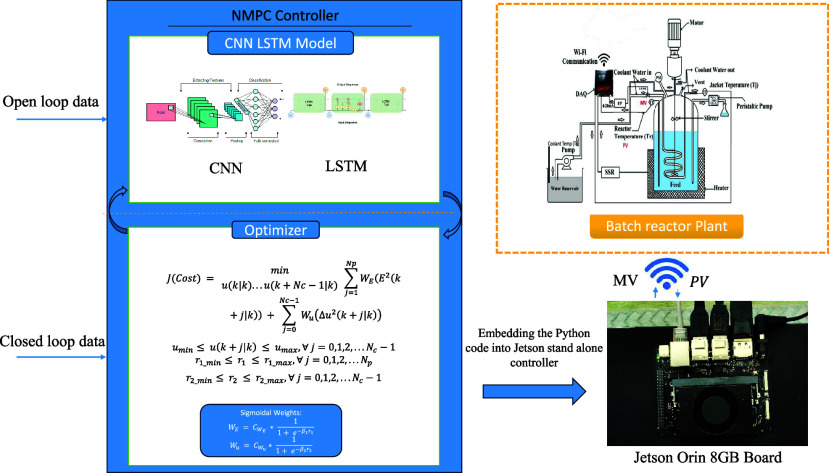

Batch reactors are type of chemical reactors, where the reactants are loaded to process for a defined batch time and the products are removed after the polymerization reaction completion. Specialty chemicals and food processing industries widely use BRs due to their versatility and suitability for handling small- to medium-scale production, complex reactions, and varying reaction conditions. This article employs a CNN-LSTM-based nonlinear model predictive controller (NMPC) to effectively track the temperature profile of a BR. This model offers significant advantages in NMPC by leveraging convolutional neural networks (CNNs) to capture spatial features and long short-term memory (LSTM) networks to manage temporal dependencies, thus enhancing prediction accuracy and control performance. The approach involves training the CNN-LSTM model using input and output data obtained from open-loop experimentation with the BR. This model evaluates the goal of optimizing the coolant flow rate while managing the heat generated by the exothermic reaction within the reactor. Additionally, a heuristic method incorporating a sigmoidal weighting functions are utilized to improve the computational efficiency of the model. The successful implementation of this CNN-LSTM-based NMPC model demonstrates its potential for large-scale usage in industrial applications. By providing accurate temperature predictions and optimizing control actions, this approach can enhance process efficiency, reduce energy consumption, and improve safety in various pharmaceutical industries.

## 1 Introduction

In process industries, the batch process is a vital and significant component. Batch processing has received more attention due to the trend toward high-quality, low-volume chemical manufacturing and enhanced operational flexibility.^1^ The versatility of the batch reactor (BR) in accommodating a diverse array of reactions and its capacity to adjust to various scales render it indispensable in both research and industry contexts. They offer advantages such as flexibility in operation, ease of scaling up or down, and better control over reaction times and conditions.

Despite their widespread use, BRs present significant challenges in terms of maintaining optimal reaction conditions, particularly when managing exothermic reactions that can lead to temperature spikes and safety risks. Traditional control methods often struggle with the nonlinear dynamics and time-dependent behavior inherent in batch processes. Control engineers have developed both linear and nonlinear control methods for the first principle-based model with random nonlinear profiles. On the other hand, some engineers have studied how to create the ideal temperature profile for a specific chemical input. During operation, the BR is initially loaded with the feed material. There is no additional input added during the process, and the product is collected at the conclusion of the batch cycle. Reactions within the BR typically involve either the release or absorption of heat.

This reactor is specifically designed for exothermic reactions, as the reaction temperature may be regulated by altering either the heater current (*H*) or the coolant flow rate (*F_t_*). Typically, the heat transfer fluid circulates through the jacket or coils to control the heat, either by adding or removing it. In order to sustain the desired temperature of the reactor contents, the following techniques can be employed.

- Adjusting the *H* while keeping the *F_t_* constant.
- Keeping the *H* constant while varying the *F_t_*.
- Employing cascade control or split range control involves managing the *F_t_* through an outer loop and varying the *H* through an inner loop.

## 2 Related Literature Review

The research has shown that this class of trajectory tracking issues responds well to model-based control strategies. When exothermic processes occur, conventional pretuned controllers are ineffective. The tracking of the reaction temperature profile is effectively achieved by utilizing model-based controllers. The ineffectiveness of traditional pretuned controllers, such as PID, is rooted in their limitations in handling the complexities of modern process plants. These controllers are typically designed for specific operating conditions and can struggle to adapt to dynamic changes, nonlinearities, and interactions between process units. As a result, their performance may degrade, leading to instability and inefficiencies in varying operational scenarios. In contrast, model predictive control (MPC) offers significant advantages by utilizing a process model to predict future behavior and optimize control actions in real-time. This capability allows MPC to manage multiple constraints and complex interactions effectively, ensuring that controlled variables remain closer to their set points. As a result, the implementation of MPC not only improves operational efficiency, but also results in energy savings and reduced equipment wear, enabling plants to operate at their maximum capacity.^2^ The MPCs excel at managing system dynamics and their ability to handle faster and more complex scenarios, even under constraints.^3^

Balakrishnan et al.^4^ investigated the incorporation of multilayer network weights into Hammerstein model blocks and the utilization of an extreme learning machine to train a feedforward network without the need for gradient calculations. These approaches simplify model identification, enabling the design of effective controllers with faster learning and reduced computational complexity. Future work proposes implementing a machine learning-based NMPC using advanced hardware.

Shettigar et al.^5^ applied wiener neural networks (WNN) for modeling and controlling BRs. The study successfully employed WNNs to track the temperature profile of a BR. The work also involved designing a generalized predictive controller (GPC) using the WNN model, which was validated experimentally to handle the nonlinearities inherent in BR temperature control effectively. This article emphasizes the role of structured neural networks in enhancing control accuracy for complex nonlinear processes. Furthering the development of nonlinear control strategies, Shettigar et al.^6^ introduced an advanced nonlinear model-based control (NMBC) model for trajectory tracking in batch polymerization reactors. The study compared NMBC with a conventional NMPC algorithm, utilizing an unscented Kalman filter (UKF) for state estimation. The results indicated that the NMBC, combined with state estimation, outperformed the NMPC in real-time control scenarios, particularly in maintaining precise temperature trajectories in batch polymerization processes. This research underscores the importance of computational efficiency in predictive control algorithms for complex industrial applications.

Differential-algebraic optimization problems (DAOPs) are an important part of process engineering. They are especially useful for improving fed-BRs, where the control problem is made more difficult by singular arcs and state variable constraints. Traditional methods often struggle with these complexities, leading to the exploration of advanced techniques like successive quadratic programming (SQP). Cuthrell and Biegler^7^ propose an innovative approach that combines SQP with orthogonal collocation on finite elements to address these challenges. SQP is favored for its robustness in solving nonlinear programming (NLP) problems, iteratively refining solutions by solving quadratic subproblems. To enhance the accuracy and stability of these solutions, orthogonal collocation has been employed, offering desirable properties for such dynamic systems. This method draws intriguing parallels to traditional variational calculus, linking NLP optimality conditions with general variational principles in optimal control. Literature shows that this combined strategy not only works well with analytically obtained solutions in easier cases, but it also gives accurate results in more complicated cases where analytical solutions are not available, like when optimizing fed-batch penicillin reactors.^8^

The tracking of the reaction temperature profile is effectively achieved by utilizing model-based controllers, such as a nonlinear cascade controller, feedback controllers, self-tuning controllers, adaptive controllers, model predictive controllers, and optimal control strategies identified from a literature survey.^9^

Lopez-Francisco^10^ created the polymerization reactor’s temperature profile. This article created a random temperature profile to be used in reactor heating. Mathematical modeling can be accomplished using two primary methods: constructing differential or partial differential equations based on the force or mass balance inside the system, or constructing the model based on input and output data. The goal of this research is to use data-driven models and predictive functionality to create an ideal controller. NMPC is commonly employed in combination with various data-driven techniques, such as autoregressive with exogenous inputs (ARX) or nonlinear ARX models, for batch and semibatch processes. However, a first-principles model can offer a more comprehensive understanding of the system’s dynamics.^11,12^

Foss et al.^13^ proposed an approach to tackle the challenge of controlling processes with wide-ranging conditions by breaking down the process into distinct operating regimes, each modeled by simple state-space structures. Interpolation combines these local models into a global model, identifying parameters through empirical data. When applied to a batch fermentation reactor, their model predictive controller outperforms both exact process and linear models, demonstrating that combining elementary process knowledge with sufficient data leads to a highly effective nonlinear model for predictive control.

Chen et al.^14^ explored the computations underlying CNNs and highlighted their broad applicability beyond image and video data. By processing grid data in various dimensions, CNNs can identify features such as patterns and geometrical properties through convolution operations. This flexibility allows CNNs to be applied in chemical engineering fields, showcasing their versatility in handling complex, nonimage data types.

Shu et al.^15^ propose a novel approach combining long short-term memory (LSTM) and convolutional neural networks (CNN) for fault diagnosis in chemical processes. The method, called multichannel LSTM-CNN (MCLSTM-CNN), first uses LSTM to capture temporal and spatial domain information from fault data. Multiple convolutional kernels then extract features through different channels, which are subsequently classified using fully connected layers. The method was tested on the Tennessee Eastman chemical process, where it outperformed several other models, demonstrating higher diagnostic accuracy and superior fault classification results.

The suggested approach utilizes a CNN-LSTM model, which has demonstrated efficacy in multiple applications, such as solar brightness predictions^16^ and stock price projections.^17^ In the CNN-LSTM model, the convolutional layers are intended to identify local patterns and structures within the nonlinear data, including short-term trends or fluctuations. The collected characteristics are subsequently transmitted to the LSTM layers, which capture long-term temporal dependencies in the data, facilitating the prediction of future positions as time series data. This method improves the model’s generalizability and robustness. It aids in the continuous endeavors within the discipline to create more thorough and dependable trajectory prediction systems.^18^

The proposed article used CNN-LSTM based implementation for the tracking. The integration of CNN with LSTM enhances predictive performance and facilitates the analysis of nonlinear and highly dynamic high-dimensional data. This is facilitated by a convolutional layer for feature extraction and a hidden layer that retains information and optimizes significant data.^19−21^

## 3 Construction of the CNN-LSTM Model

A hybrid architecture of convolutional neural networks (CNN) and long short-term memory (LSTM) networks, commonly known as CNN-LSTM, is used in the proposed model to effectively capture both spatial and temporal features from the input data. The data for constructing the CNN-LSTM model is collected using the extensive open-loop implementation of the BR that captures features such as reactor temperature (*T*_r_), coolant flow rate (*F_t_*), jacket temperature (*T*_j_), and heater current (*H*). The *T*_r_ is the state *x*, and the *F_t_* is the input *u*. These open-loop simulations were performed, collecting 10,300 examples for a small-time step. For a BR, it is difficult to describe the change accurately, so an extended sliding window transforms the collected time series data into dynamic data. After the transformation, the data is split into training and test data sets.^22,23^

Adjusting *F_t_* according to *T*_r_ is sufficient to maintain the heat caused by the exothermic reaction in the BR. Thus, a multiple input and single output CNN-LSTM model that predicts the *T*_r_ is proposed. The CNN-LSTM model is trained to predict the one-time step-ahead *T*_r_ at *x* (*t* + 1) using the present and past *T*_r_ at *x* (*t* = 0, 1, ...l) and coolant flow rate at *u (t* = 0, 1, ...l) inputs. The time lag *l* is determined through experiments.^24^

### 3.1 Structure of the CNN-LSTM Model

The model starts with an input layer that accepts data with the shape (*None*, 100, 2). The first layer is a one-dimensional convolution layer (*Conv*1D) with 32 filters and a kernel size of 3, producing an output shape of (*None*, 100, 32). Equation 1 represents the convolutional layer.
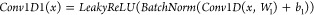
1

*Conv*1*D* denotes a 1D convolution operation, where *x* is the input, *W*_1_ is the convolutional kernel (*W*_1_), and *b*_1_ is the bias term. The output *z* in eq 2 is a feature map resulting from the convolution operation.
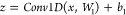
2

Equation 3 represents the output of the convolution operation at position *t*, which is *z*[*t*]. The kernel’s length is denoted by *K*, *x*[*t* + *k*] is the input at position *t* + *k*, *W*_1_[*k*] is the weight of the kernel at position *k*, *b*_1_ is the bias term added to each position of the output.
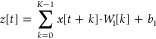
3

### 3.2 Batch Normalization

This process normalizes the output and then scaling and shifting it using learned parameters. Batch normalization is given in eq 5.
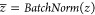
4
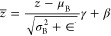
5

Here, μ_B_ is the mean and σ_B_^2^ is the variance of the batch, γ, β are the learnable scale and shift parameters, and ∈ is a constant for numerical stability. After, batch normalization is applied to the convolutional output to standardize the activations and improve the network’s stability. The normalized output shape remains (*None*, 100, 32).

### 3.3 Leaky ReLU Activation

After applying a *LeakyReLU* activation function to incorporate nonlinearity into the model, a max pooling layer (*MaxPooling1D*) with a pool size of 2 is used. This reduces the output shape to (*None*, 50, and 32). The *LeakyReLU* activation function is given in eq 6.
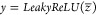
6

The second convolutional layer (*Conv1D*) has 64 filters and a kernel size of 3, producing an output shape of (*None*, 50, 64). This is given in eq 7.
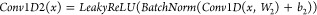
7

Batch normalization and *LeakyReLU* activation are again applied, followed by another max pooling layer (*MaxPooling1D*) with a pool size of 2, resulting in an output shape of (*None*, 25, 64).

The third convolutional layer (*Conv1D*) also has 64 filters and a kernel size of 3, producing an output shape of (*None*, 25, 64). Equation 8 represents this layer.
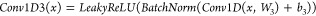
8

Batch normalization and *LeakyReLU* activation are applied, followed by a final max pooling layer (*MaxPooling1D*) with a pool size of 2, resulting in an output shape of (*None*, 13, 64).

A dense layer with 512 units is then applied to the flattened output of the last max pooling layer, resulting in an output shape of (*None*, 13, 512). The eq 9 represents the dense layer.
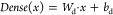
9

Following the dense layer, an LSTM layer with 1024 units is used to capture the temporal dependencies in the data. The LSTM cell includes several gates that regulate the flow of information.

1. **Forget Gate:** The forget gate function is given in eq 10
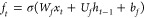
10
2. **Input Gate:** The input gate operation is given in eq 11
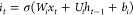
11
3. **Cell State:** The cell state operation is given in eqs 12 and 13

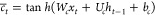
12
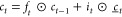
13

4. **Output Gate:** The output gate operation is given in eqs 14 and 15

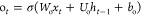
14
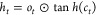
15

Finally, the output from the LSTM layer is fed into a dense layer with a single unit and a linear activation function, producing the final output. This can be represented as given in eq 16.
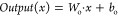
16

The CNN-LSTM models are trained using Python’s Keras Deep Learning Library. The model’s architecture is determined through experimentation. An adaptive moment estimation approach solves an optimization issue that minimizes the modeling error during the training phase.^25^

## 4 Nonlinear Model Predictive Controller (NMPC) Design

The NMPC model predicts future outcomes within a limited number of time steps. The fundamental objective is to achieve an ideal *F_t_* while preserving the *T*_r_. Consequently, the NMPC model guarantees an appropriate discharge of coolant from the BR. The NMPC model utilizes historical *T*_r_, *F_t_*, and the CNN-LSTM model to determine the prescribed *F_t_*. The soft constraint strategy is employed to regulate the manipulated variable (*u*). The objective function is minimized by include the slack variables, which in turn relaxes the constraints.^26^

For time step *t*, a fixed prediction horizon of process variable, and the fixed control horizon, the scheduler is formulated as given in eq 17.
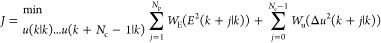
17

Equation 17 uses the values of *E*(*k* + *j*|*k*) and Δ*u*(*k* + *j*|*k*) given in eqs 18 and 19.
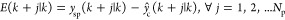
18
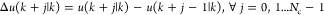
19with subject to constraints
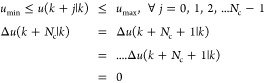


### 4.1 Heuristic Methods: Sigmoid Functions

Nonlinear Model Predictive Control (NMPC) is renowned for its capability to manage complex, nonlinear systems with constraints. However, this capability comes at the expense of significant computational costs. NMPC necessitates solving nonlinear optimization problems at each control interval, which involve predicting future system states over a defined horizon and optimizing control actions while adhering to constraints. This process can be computationally intensive, particularly for systems with high-dimensional state and control spaces, leading to long computation times and high resource consumption.^11^

Heuristic enhancements are employed to improve efficiency and mitigate the computational challenges associated with NMPC. One such enhancement involves the application of the sigmoid function. The sigmoid function defined as  produces smooth, continuous nonlinearity into the NMPC framework, which offers several advantages:

1. **Simplified Constraint Handling**: The sigmoid function can approximate or smooth nonlinear constraints, transforming them into bounded and continuous forms. This simplification reduces the complexity of the optimization problem and facilitates more efficient solution processes.
2. **Smooth Approximation**: Using the sigmoid function, piecewise linear or nonsmooth components of the objective function and constraints can be approximated with smoother, continuous functions. This smoothness aids in achieving faster and more stable convergence of the optimization algorithms.
3. **Enhanced Computational Efficiency**: The application of the sigmoid function streamlines the evaluation of complex, nonlinear relationships within the NMPC framework. This enhancement results in a reduced computational load and faster calculation times, making the NMPC system more suitable for real-time applications.

Without heuristic enhancements, NMPC computation involves handling intricate, time-consuming optimization problems that may lead to numerical instability and prolonged solution times. The introduction of the sigmoid function as a heuristic approach simplifies these problems by smoothing constraints and objectives, thereby improving computational efficiency. This results in shorter computation times and better real-time performance, demonstrating the effectiveness of the sigmoid function in reducing NMPC’s computational expense.

Nonlinearity in MPC offers significant advantages over fixed-weight approaches, particularly in tracking performance. Adaptive tracking MPC schemes can effectively manage unknown nonlinear systems, demonstrating improved tracking capabilities compared to traditional fixed-weight methods. Fixed weights often limit performance in dynamic environments, while nonlinear MPC adapts more effectively to changes, leading to better handling of system dynamics.^27^ Conventional weights of *w*_e_ and *w*_u_ are given in eqs 20 and 21
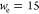
20
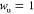
21

In the objective function, the output error weights *W*_E_ and the control input weight *W*_u_ are replaced by the sigmoid function as given in eqs 22 and 23
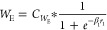
22
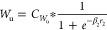
23

Here β_1_ and β_2_ are the slopes of sigmoid functions *W*_E_ and *W*_u_, respectively. The arguments *r*_1_ and *r*_2_ are real numbers. For time step *t*, a fixed prediction horizon, and the fixed control horizon, the scheduler is formulated as given in eq 24.
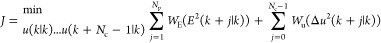
24

Subject to
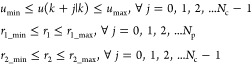


### 4.2 Batch Reactor Dynamics

**Figure 1:**
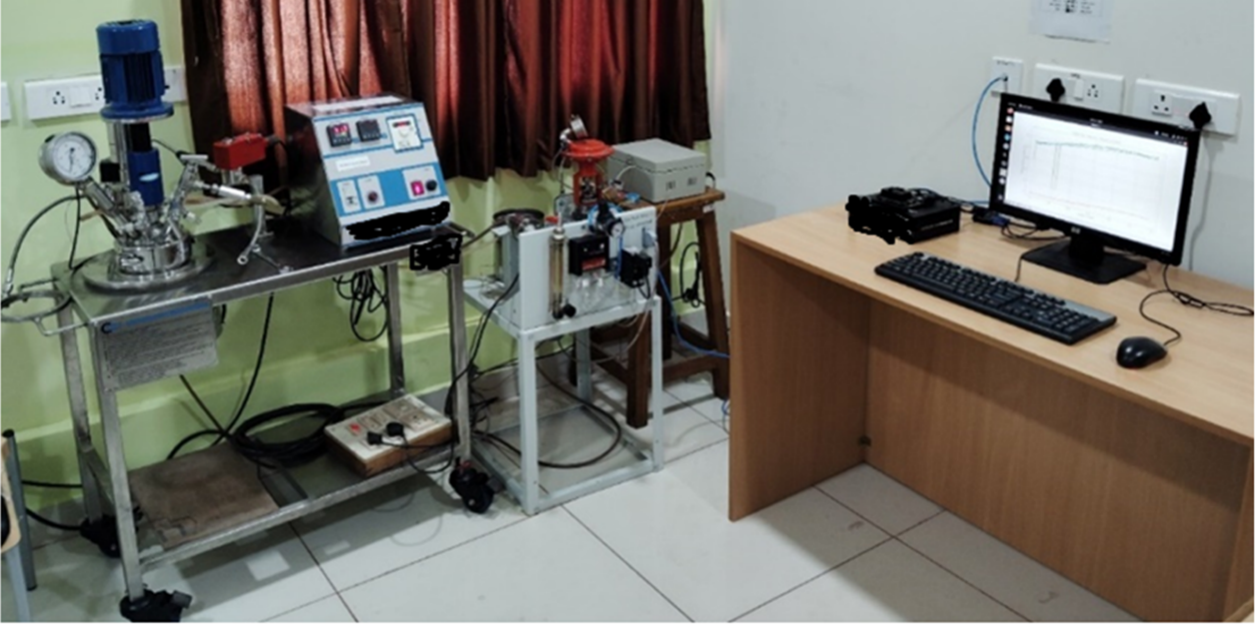
Pilot plant BR setup available in the Machine Learning for Advanced Process Control lab, ICE Dept., MIT, Manipal. Image captured by Thirunavukkarasu Indiran.

**Figure 2:**
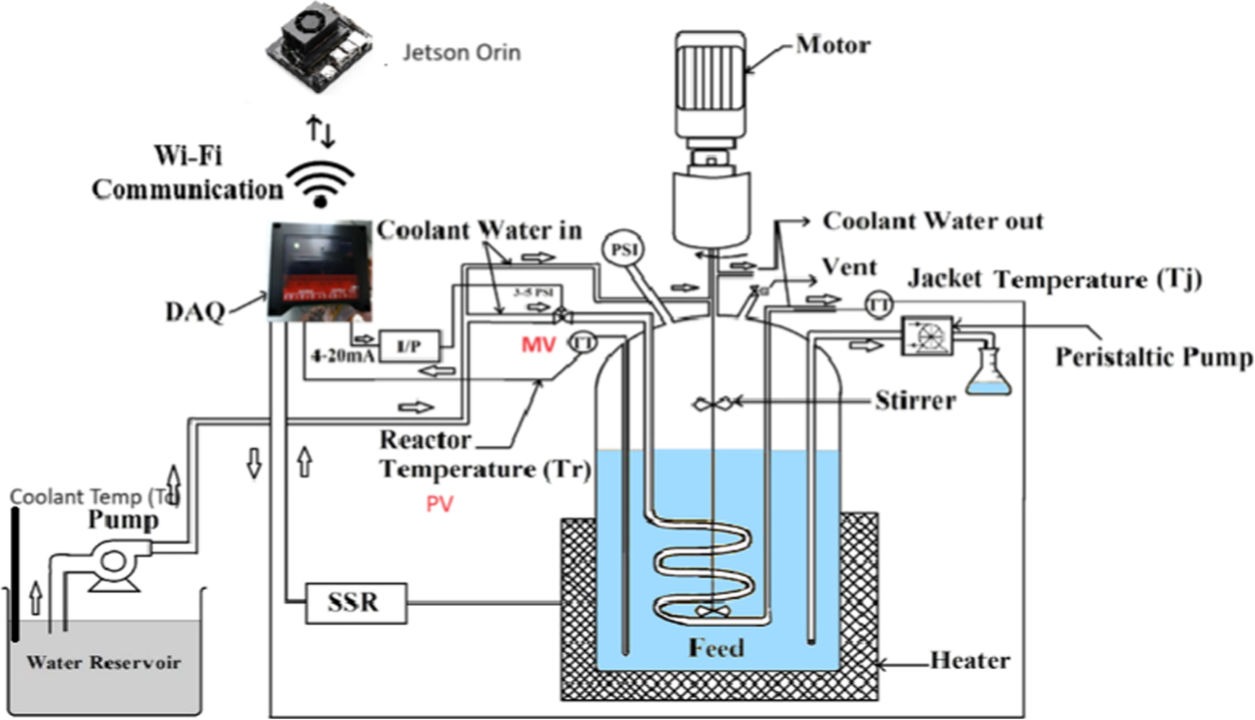
Schematic of the batch reactor with Jetson Orin for embedding the Python code for validation. Image developed by Thirunavukkarasu Indiran.

The pilot plant BR system consists of a reactor vessel that is fitted with a heater coil, a coolant flow coil, and a stirrer. This configuration is illustrated in Figure 1. The Piping and Instrumentation diagram for this BR is shown in Figure 2. The reactor has a capacity of 1 L, with a heater rated at 1500 W, operating on a single phase at 230 V, and a coolant flow capacity of 0.75 L per minute when the valve is fully open. The stirrer’s speed is set at 200 rpm for this study, although it can reach a maximum of 1500 rpm. The experiments use an HP desktop with 8GB of RAM, an i5-6500 processor running at 3.02 GHz, a 64-bit operating system, and MATLAB 2019b. The experiment employ water as the heat transfer medium, which flows through the coils to disperse heat.

The system dynamics of the BR include the energy balance of the jacket, as well as the equations for reaction kinetics. This study employs an acrylamide polymerization reaction, where the amounts of the initiator ammonium persulfate [I] and the monomer acrylamide [M] are estimated^4^ and provided in eqs 25 and 26.
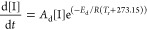
25
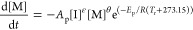
26

Equations 27 and 28 provide the dynamics of *T*_r_ and *T*_j_.
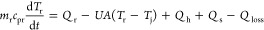
27
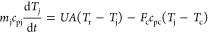
28

The values for *Q*_r_, *Q*_s_, *Q*_loss_, *m*_r_*c*_pr_, *and m*_j_*c*_pj_ are calculated from eqs 29, 30, 31, 32, and 33 respectively, and then used in eqs 25 and 26.
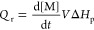
29
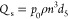
30
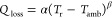
31
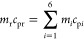
32
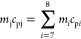
33

The heat loss coefficients α and β are determined by a least-squares method, whereas the overall heat transfer coefficient *U* is calculated based on the time constant of the BR.

### 4.3 Forecasting Ability of the Proposed LSTM Model

**Figure 3:**
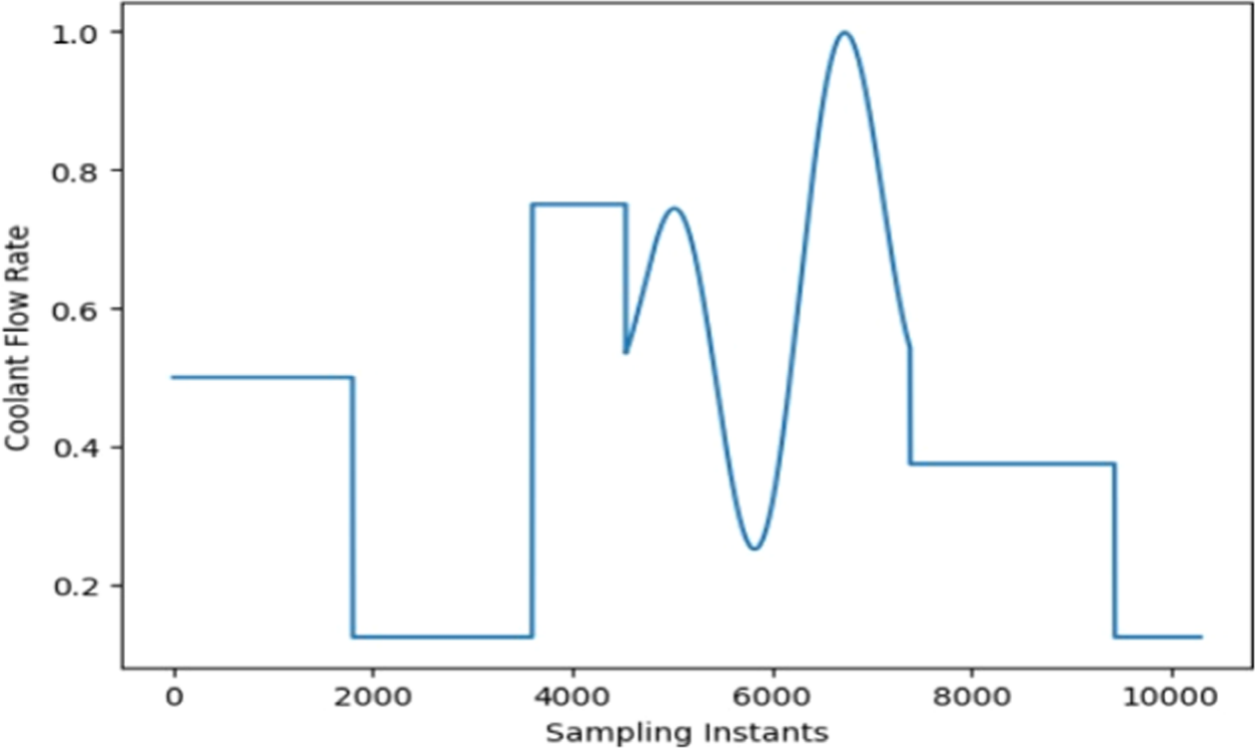
Plot of coolant flow rate used to collect the *T*_r_.

**Figure 4:**
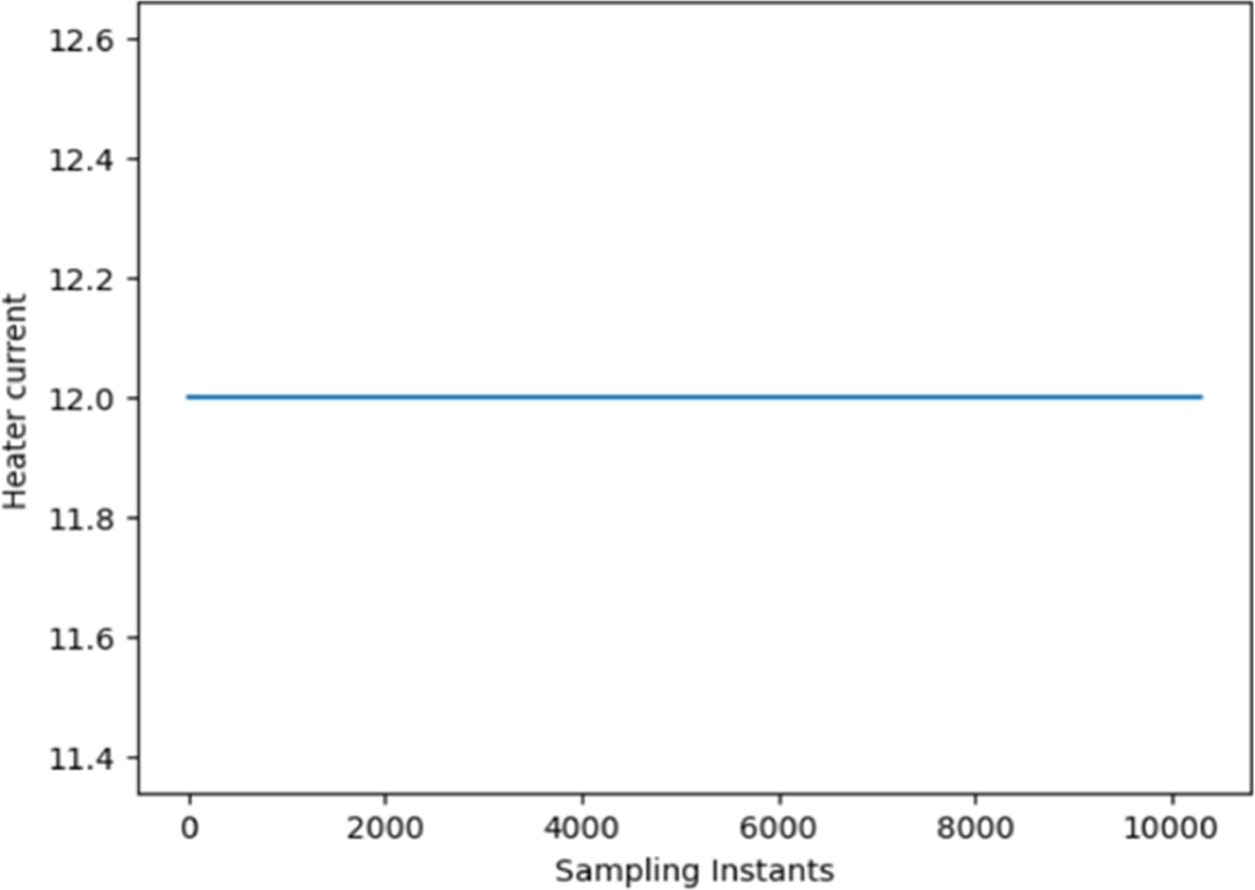
Heater supply controller in-terms of 4–20 mA signal using Solid State Relay.

**Figure 5:**
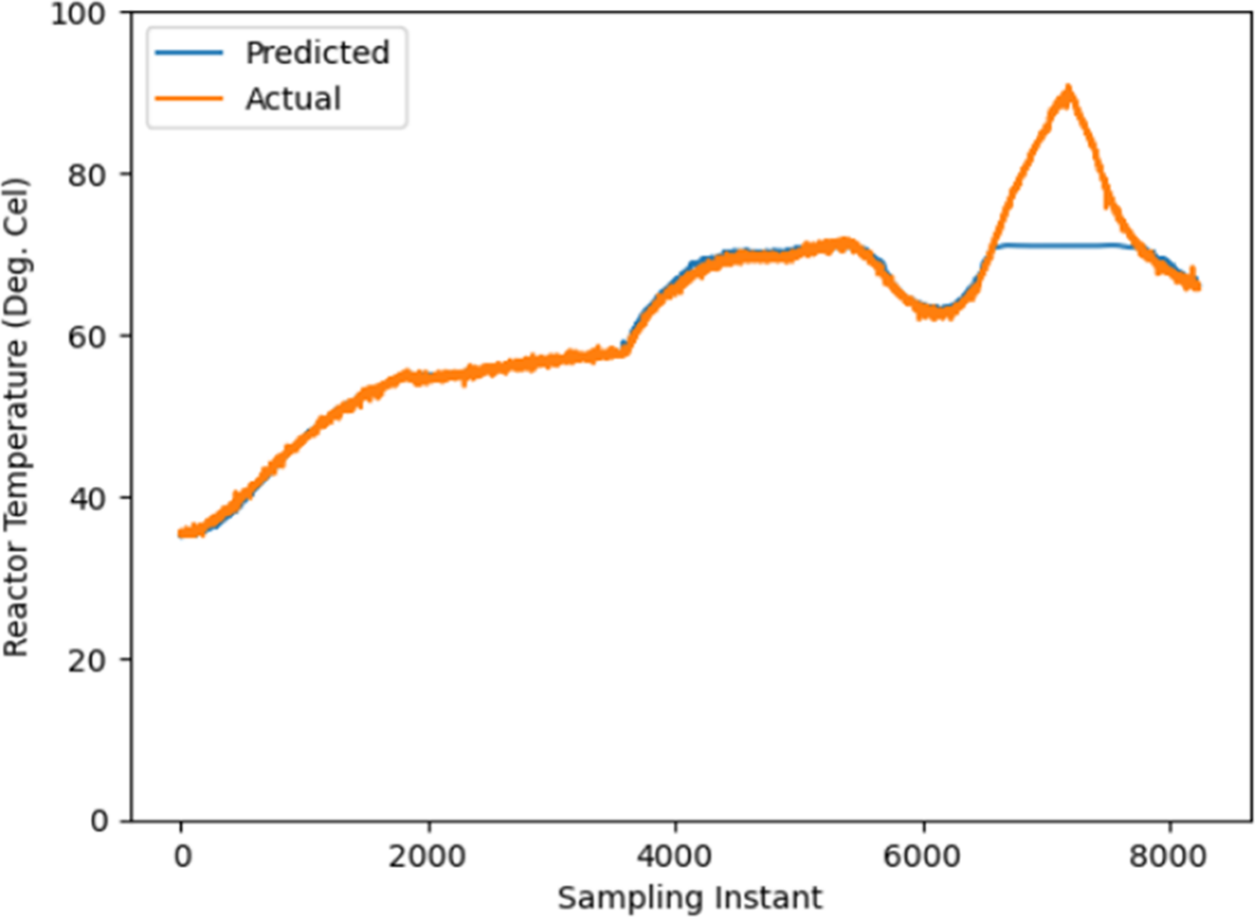
Model fit obtained using CNN-LSTM using input output data.

The open-loop data on *T*_r_ is collected by varying the coolant flow rate as shown in Figure 3 and keeping the heater supply constant for 10,300 samples as shown in Figure 4. The CNN-LSTM model utilizes the collected data to model BRs, as shown in Figure 5. The forecasting capacity of the proposed model was evaluated by using a training sequence length of 30 days without any lag. The training process consisted of 20 epochs. The proposed article chose to train the model for 20 epochs based on our empirical observations during experimentation. After 20 epochs, the model’s training loss reached a stable point, and the performance on the testing data was optimal, with minimal loss. Continuing training for more epochs could have resulted in overfitting, where the model would become too tailored to the training data and lose generalization capacity. This article prioritized avoiding overfitting to ensure the model’s ability to perform well on new, unseen data.

**Figure 6:**
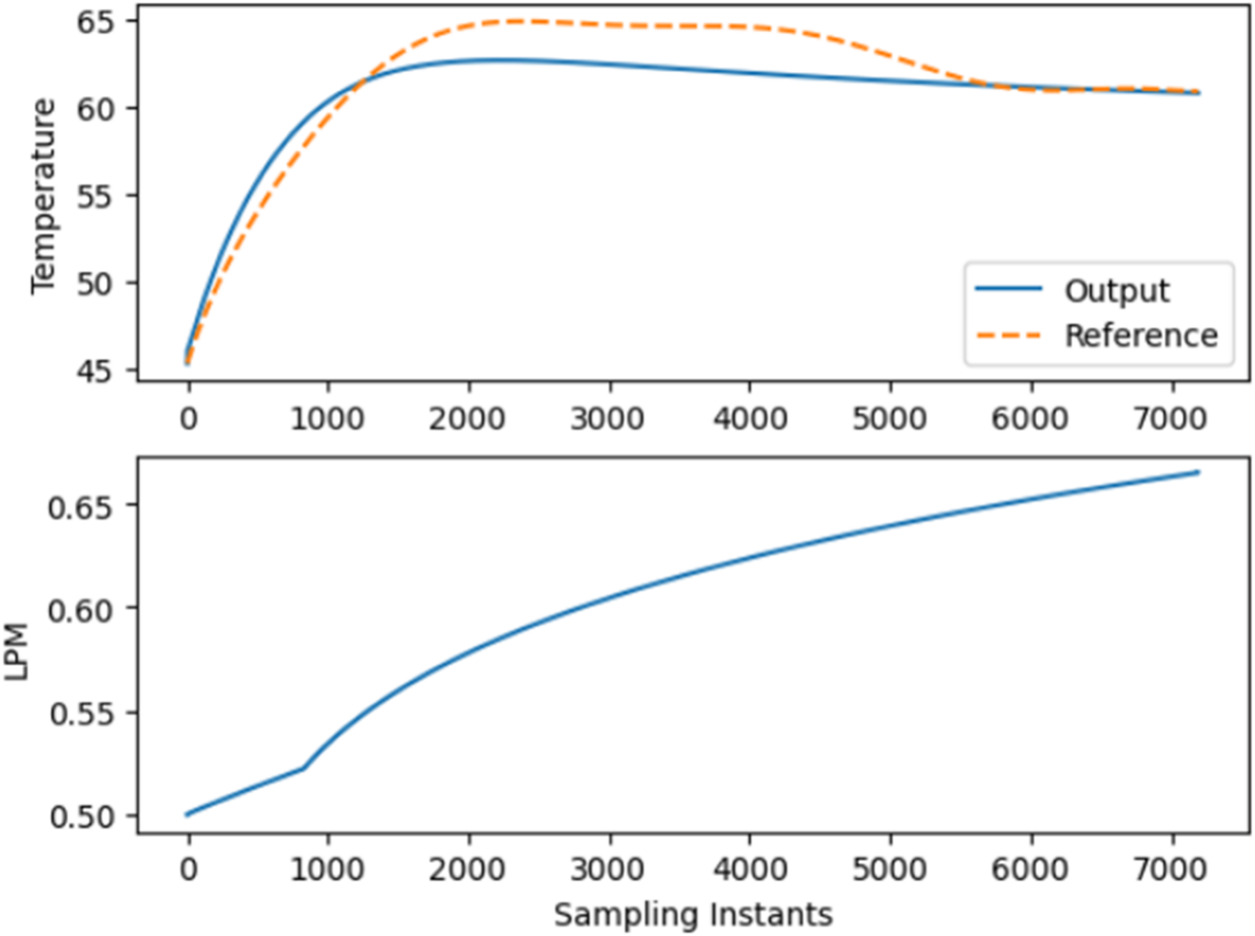
Closed-loop trajectory tracking using CNN-LSTM NMPC “*P*_NMPC_(*x*)” using constant weights.

**Figure 7:**
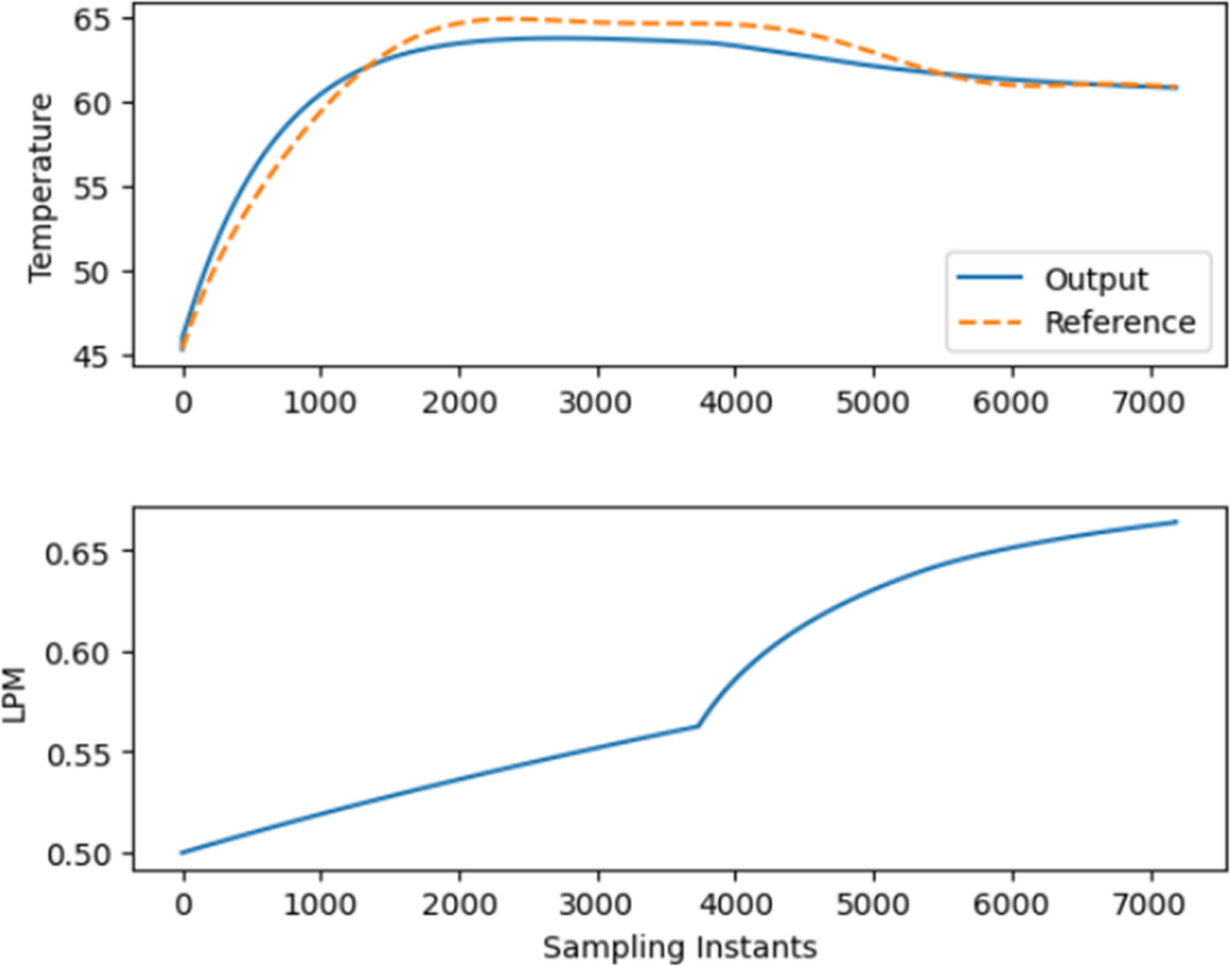
Closed-loop trajectory tracking using CNN LSTM NMPC “*P*_SIG_(*x*)” sigmoidal weights.

Figures 6 and 7 illustrate the use of NMPC models *P*_NMPC_(*x*) and *P*_SIG_(*x*) to forecast the *F_t_* for the BR. Tables 1 and 2 display the hyperparameters utilized in both models *P*_NMPC_(*x*) and *P*_SIG_(*x*). The *F_t_* has lower and upper bounds of 0 and 1.5, respectively. The lower and upper bounds of *r*_1_ are between 15 and 50, respectively; for *r*_2_, the bounds are between 5 and 10. The slop value of the sigmoid function β_1_ is 0.001, whereas that of β_2_ is 0.095. The prediction horizon *N*_p_, which is the future time period over which predictions are made by a forecasting model, is given a value of 10, and the control horizon *N*_c_, which is the future time period over which control actions are optimized in a model predictive control system, is provided a value of 8.

**Table 1:** Hyperparameters of *P*_NMPC_(*x*)

tuning parameters	values
*W*_E_	15
*W*_u_	1.2
*u*_min_	0.001
*u*_max_	1.5
*N*_p_	10
*N*_c_	8

**Table 2:** Hyperparameters of *P*_SIG_ (*x*)

tuning parameters	values
*N*_p_	10
*N*_c_	8
β_1_	0.001
β_2_	0.095
*r*_1 min_	15
*r*_1 max_	50
*r*_2 min_	5
*r*_2 max_	10
*C*_*W*_E__	15
*C*_*W*_u__	3

As seen in Figures 6 and 7, the tracking of temperature with respect to the desired trajectory of the proposed, highly nonlinear CNN LSTM based NMPC is better compared to the conventional constant weights used so far.

Table 3 shows the performance index of the NMPC using conventional weights and sigmoidal weights. Table 3 indicates a significant improvement in error metrics between the two control schemes, *P*_NMPC_(*x*) and *P*_SIG_(*x*) specifically, the Mean Squared Error (MSE), Mean Absolute Error (MAE), and Integral MSE for *P*_SIG_(*x*) are all lower than those for *P*_NMPC_(*x*).

**Table 3:** Performance Index

s. no.	MSE	MAE	integral MSE
*P*_NMPC_(*x*)	2.6674	1.5515	2.6674
*P*_SIG_ (*x*)	1.0038	0.9798	1.0038

The MSE for *P*_SIG_(*x*) is 1.0038, compared to 2.6674 for *P*_NMPC_(*x*), indicating that the error has decreased by approximately 1.6636. Similarly, the MAE for *P*_SIG_(*x*) is 0.9798, which is about 0.5717 less than the 1.5515 for *P*_NMPC_(*x*). This reduction in error signifies that the *P*_SIG_(*x*) approach demonstrates improved accuracy and performance in controlling the system compared to *P*_NMPC_(*x*), effectively enhancing the overall control quality.

It is evident that the sigmoidal NMPC performance is better than the conventional NMPC performance, since the integral performance measure values of sigmoidal weights are much lesser than the conventional NMPC.

### 4.4 Real-Time Validation

**Figure 8:**
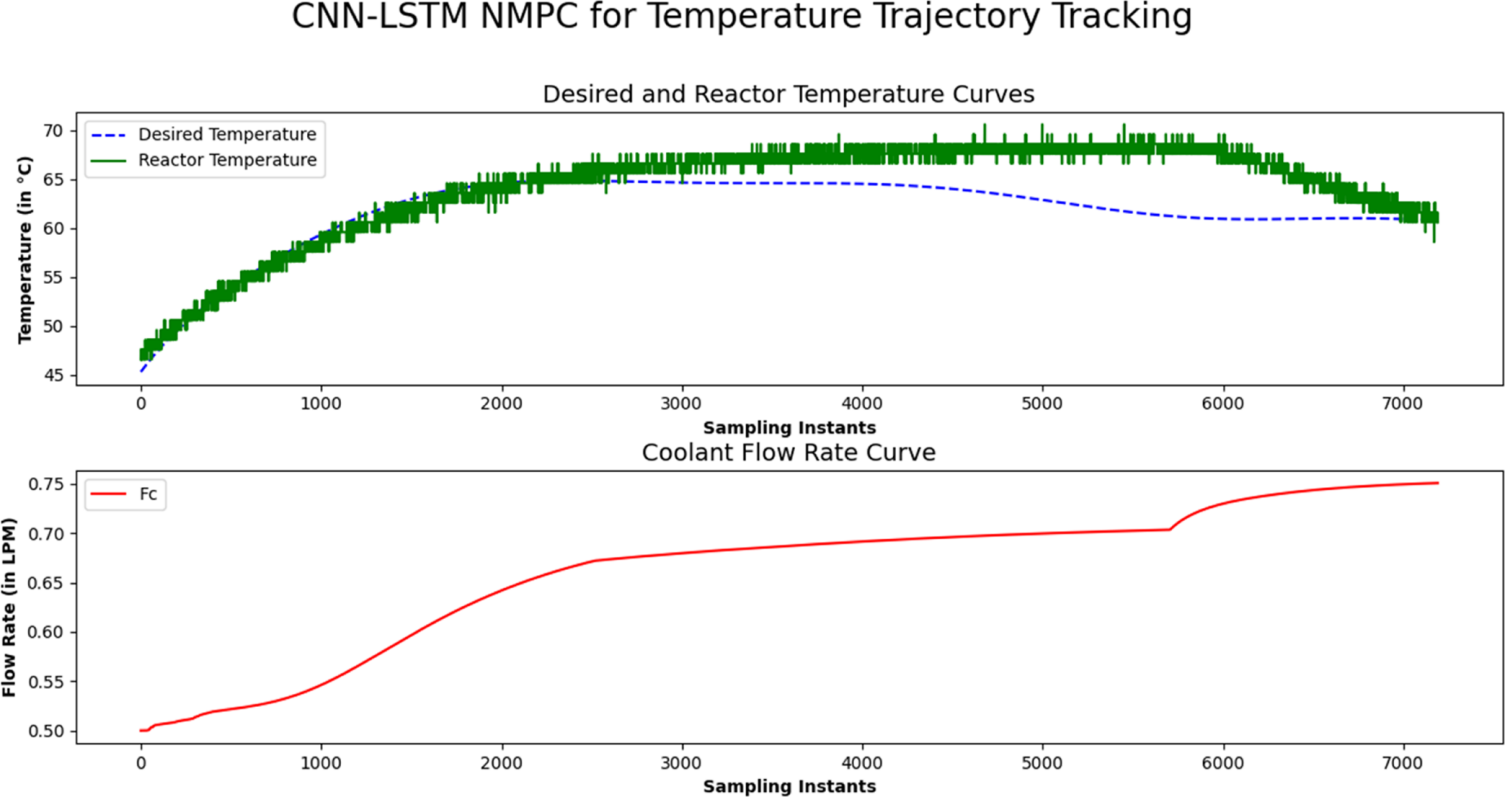
Experimental closed-loop validation of CNN-LSTM based NMPC on a lab-scale Batch Reactor available at Machine Learning for Advanced Process Control Lab, MIT, Manipal via Python code using Jetson Orin 8GB board.

The pilot plant BR setup available in the Machine Learning for Advanced Process Control lab, ICE Dept., MIT, Manipal, shown in Figure 1, was used for the data collection for the real time validation. Figure 8 illustrates the closed-loop real-time trajectory tracking using CNN LSTM NMPC on a laboratory-scale batch reactor using Jetson Orin. The CNN-LSTM model was developed using the input output data collected from the open-loop response using Python code. The predicted reactor temperature from the CNN LSTM model was further used with the NMPC for the control signals. During the experimental validation of NMPC, the control signal was active to bring down the reactor temperature with the help of sigmoidal weighting function. The coolant flow rate is also smooth without any aggressive nature with the sigmoidal weights compared to the constant weights in NMPC.

## 5 Conclusions

This article presents the development of a highly nonlinear batch reactor system utilizing a CNN-LSTM network. This model utilizes the open-loop data obtained from the batch reactor. A Nonlinear Model Predictive Controller is formulated by employing a nonlinear sigmoidal weighting function in place of conventional constant weights. The utilization of a nonlinear weighting function exhibits superior tracking performance in relation to the target trajectory when compared to the NMPC employing a constant weighting function. This article proposes that researchers working on NMPC should experiment with a nonlinear weighting function to achieve improved outcomes specially during the experimental validation.

## Notes

In near future, there are plans to produce biodiesel in a batch reactor. The best temperature profile will be established to maximize biodiesel production and to minimize the byproduct. The optimal temperature profile for the biodiesel in batch reactor is already optimized. The suggested NMPC controller discussed in this research can be utilized to validate the closed-loop operation of the reinforcement learning using the Jetson Orin 8GB board.
